# Multi-Locomotion Design and Implementation of Transverse Ledge Brachiation Robot Inspired by Sport Climbing

**DOI:** 10.3390/biomimetics8020204

**Published:** 2023-05-16

**Authors:** Chi-Ying Lin, Jhe-Ming Lee

**Affiliations:** Department of Mechanical Engineering, National Taiwan University of Science and Technology, No. 43, Keelung Rd., Sec. 4, Taipei 106, Taiwan; a25896365@gmail.com

**Keywords:** sport climbing, transverse ledge brachiation, ricochetal brachiation, multi-locomotion design, parallel four-link posture

## Abstract

Brachiation robots mimic the locomotion of bio-primates, including continuous brachiation and ricochetal brachiation. The hand-eye coordination involved in ricochetal brachiation is complex. Few studies have integrated both continuous and ricochetal brachiation within the same robot. This study seeks to fill this gap. The proposed design mimics the transverse movements of sports climbers holding onto horizontal wall ledges. We analyzed the cause-and-effect relationship among the phases of a single locomotion cycle. This led us to apply a parallel four-link posture constraint in model-based simulation. To facilitate smooth coordination and efficient energy accumulation, we derived the required phase switching conditions as well as joint motion trajectories. Based on a two-hand-release design, we propose a new style of transverse ricochetal brachiation. This design better exploits inertial energy storage for enhanced moving distance. Experiments demonstrate the effectiveness of the proposed design. A simple evaluation method based on the final robot posture from the previous locomotion cycle is applied to predict the success of subsequent locomotion cycles. This evaluation method serves as a valuable reference for future research.

## 1. Introduction

Transverse ledge brachiation robots are bio-inspired climbing robots that mimic climbing athletes [1,2,3]. They move sideways across walls by grabbing wall protrusions [4], swinging their lower limb(s), and using alternating handholds. Such robots can take the place of humans in hazardous work such as monitoring, maintenance, and rescue [5,6,7,8,9] in situations involving wall ledges such as rain deflectors, exterior window sills or trim, flat/pitched roofs [10], or friezes. For instance, densely-populated urban building areas are excellent implementation venues for transverse ledge brachiation robots.

Transverse ledge brachiation robots first swing their lower limb(s) and body to accumulate adequate kinetic energy. They then release one hand to grab the target ledge while simultaneously adjusting their posture so that the other hand can return to the original double handhold position under the guidance of inertia. This facilitates the beginning of swing excitation in the next locomotion cycle. As the processes of energy storage/release repeat and alternate, the robot traverses along the wall [11]. This unique locomotion gait is called transverse ledge brachiation (TLB). In events where swift locomotion is needed, or the distance between climbing ledges is greater than the robot’s arm span [12,13], climbers can let go of the ledge with one hand “in the instant before” the other hand grabs the target ledge. This results in a leap during which neither hand is in contact with a ledge. This completely releases the energy that the robot has accumulated during the swinging process to obtain better locomotion efficiency. This type of locomotion is called transverse ledge ricochetal brachiation (TLRB) [1].

Humans and apes are both primates, so brachiation robots modeled on humans and apes are similar [14]. However, the target handholds of ape-inspired robots are round ladder rungs [15,16] or cables [17,18], whereas TLB robots move transversely at 90° to ledges [2]. Conventional brachiation robots mostly focus on efficiently accumulating energy during the swinging process and planning joint trajectories to facilitate continuous brachiation between spaced rungs [19,20,21]. However, in addition to the swing actions, special consideration must also be given to the grasping action during the gait process of ricochetal brachiation [22,23]. The complexity of the required hand-eye coordination [1,2] means research on such robots is rare. Even fewer studies include comprehensive practical assessments.

Transverse brachiation requires good multi-limb coordination [24]. A simple two-link brachiation robot [12,13,15,17,18,19] is only capable of performing status switches between the swing phase and the grabbing phase [25]. Furthermore, fulfilling the grabbing conditions of rectangular ledges is not as easy as that of round bars. Impact dynamics can make it difficult to maintain a good grip posture when grabbing a ledge [1]. It is also difficult to perform three-dimensional posture compensation in robots with a low degree of freedom (DOF) in their actuators.

Most existing studies of transverse brachiation robots used multi-limbed robot designs based on human climbing motions [20,26,27,28]. Lin and Yang [1] employed a four-link arm-body-tail robot configuration, dividing transverse ricochetal brachiation into a swing phase, a flight phase, and a landing phase. During the leap process, or “flight phase,” they used a posture compensation control algorithm to maintain a vertical body posture. This means both arms can grab horizontal ledges smoothly while obtaining longer flight time and longer grasping distance. In addition, using a four-link arm-body-tail robot configuration, Lin and Tien [2] designed a transverse brachiation locomotion gait for situations involving horizontal ledges with large spans or different elevations. Their design includes the following four phases: release, body reversal, swinging up, and grasping. However, due to mechanical hardware limitations, their robot could only complete one locomotion cycle with this gait. Drawing inspiration from the climbing motions of athletes with greater muscle strength, Lin and Hu [10] proposed a five-DOF design under the premise of relying only on upper body strength (i.e., no lower limbs or tail); the design included a body, grippers, and shoulders and had a locomotion gait that could climb back and forth along a ledge. We refer to this gait as transverse ledge climbing. Their locomotion control strategy is based on the central pattern generator (CPG) algorithm and infrared sensors. It successfully achieves continuous climbing on horizontal and sloped ledges. However, this transverse climbing motion is not ergonomic, nor is it a type of brachiation; thus, it is more suited for situations where the lower limbs cannot swing.

Table 1 compares studies on transverse ledge climbing/brachiation robots. As can be seen, the primary limitations of existing studies include (1) a single locomotion gait and (2) the inability to perform multiple cycles. The final posture of a robot’s grippers as it transitions from the swing phase to the grabbing phase is inevitably affected by the motions of the previous phase. Thus, a non-negligible offset accumulates, which means the pendulum-like boundary conditions of the next locomotion cycle cannot be met. An inadequate grip affects system excitation and the subsequent movement phases, thereby preventing a continuous brachiation gait (via swing). Another crucial factor neglected by existing studies is the influence of the motions in each phase on those in the next phase, which affects the smoothness of transitions. Athletes are characterized by their ability to make graceful movements that exploit inertial energy [29]. In the event of external disturbances, motions are likely to become uncoordinated to the point of gait failure.

In this study, we refer to the unique movements of athletes climbing transversely along wall ledges to propose a design process that takes the cause-and-effect relationships in each phase of movement into account. This process enabled us to realize multiple locomotion gaits for coordinated bio-inspired robots. We used a parallel four-link posture as the constraint. Using model-based dynamic analysis, we derived the design parameters and joint trajectories for smooth transitions. Experiments demonstrate the efficacy of the proposed gait design method. Based on these results, we propose an evaluation index to analyze whether the grip posture of the grippers meets the boundary conditions of the swing phase to predict the success rate of achieving the next locomotion cycle. This evaluation tool serves as a valuable reference for future research.

The remainder of this paper is organized as follows. Section 2 presents locomotion analysis and classified phases for the two types of brachiation. Section 3 summarizes the dynamic modeling results used for multi-locomotion design. Section 4 offers an analysis of phase switching parameters and required joint trajectories for a smooth transition. Section 5 presents the transverse brachiation experiments based on a four-link arm-body-tail configuration robot. Finally, concluding remarks are made.

## 2. Locomotion for Transverse Ledge Brachiation

In this section, we discuss the locomotion gaits associated with continuous transverse brachiation and hybrid transverse brachiation. Specifically, we explore design requirements based on the movement phases. The complete gait design process is presented in subsequent sections.

### 2.1. Transverse Ledge Brachiation Based on Athletes’ Locomotion

**Figure 1:**
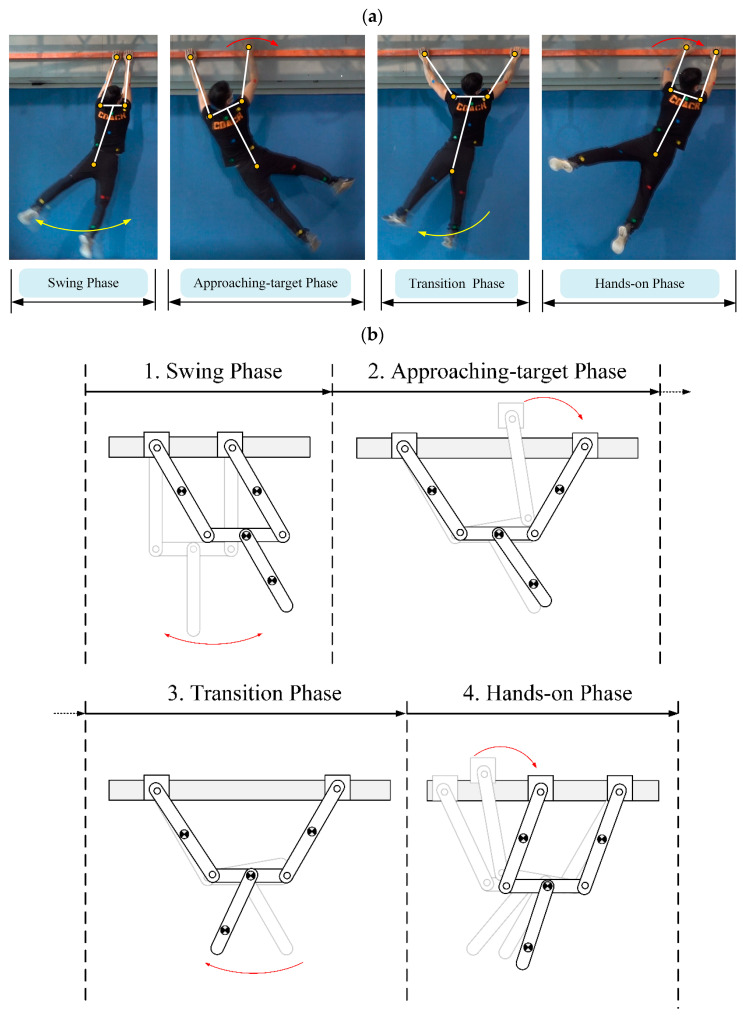
Phase classification for transverse ledge brachiation: (**a**) locomotion analysis of a sport climber [11]; (**b**) robot locomotion design. Yellow and red arrows indicate moving direction.

Sport climbers transverse brachiate using a periodic transverse movement behavior [11]. Figure 1a dissects this locomotion mode. First, the athlete swings his lower limbs to increase system energy and the overall swing range. Once enough energy has been accumulated; the athlete is able to grasp the ledge with his right hand. In this phase, the system energy is converted from kinetic to potential energy. We found that the athlete does not grasp the ledge as far as possible but maintains a suitable distance between his hands. This strategy is critical to whether subsequent movement phases can be completed successfully. After his right hand has grasped the wall ledge, the swinging of the lower limbs rotates the upper limbs and torso, which adjusts the athlete’s posture. Finally, his left-hand grasps the ledge to the right, completing a single transverse brachiation locomotion cycle. Based on the gait analysis above, we can divide the locomotion gait into the four phases shown in Figure 1b: a swing phase, a target-approaching phase, a transition phase, and a hands-on phase. Each phase must be completed, as shown in Figure 1a, in order to enter the next phase. If any movement in a phase is incorrect or if the timing of phase switching is off, then the motion will immediately become uncoordinated and cause the locomotion gait to fail. The most common consequence is that the athlete will fall to the ground. This outcome is unacceptable for a ledge-climbing robot. Thus, in this study, we focus on the means of planning the motion processes of each phase and the postures needed for the robot to transition from one movement phase to the next smoothly.

### 2.2. Transverse Ledge Ricochetal Brachiation Based on Athletes’ Locomotion

**Figure 2:**
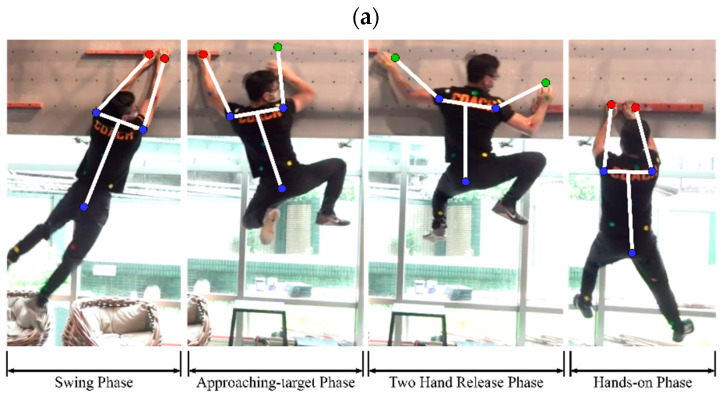

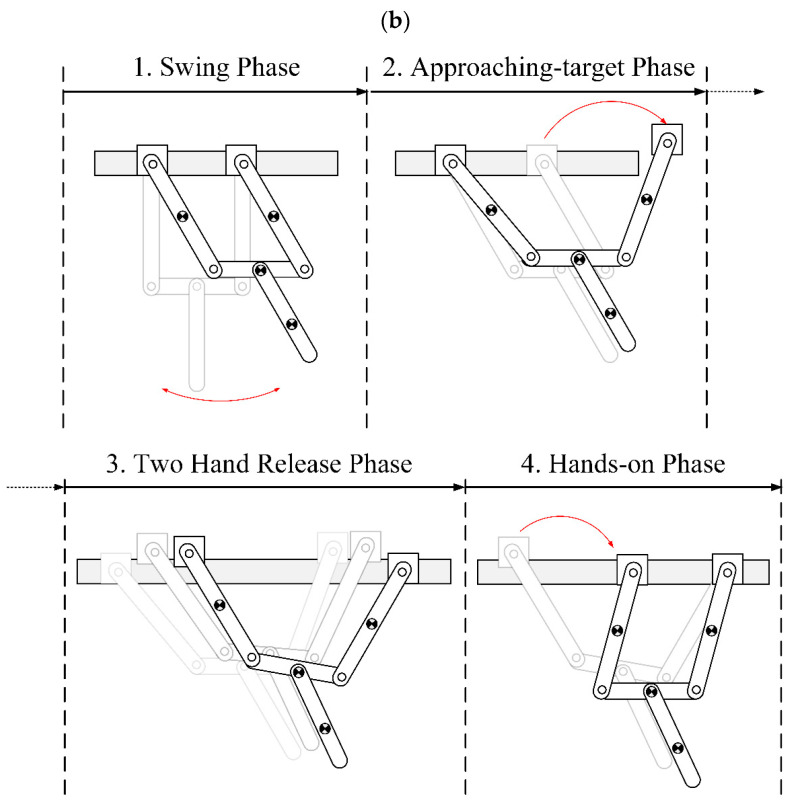
Phase classification for transverse ledge ricochetal brachiation: (**a**) locomotion analysis of climbing athlete where red indicates holding, green indicates releasing and blue indicates the actuating joints [11]; (**b**) robot locomotion design.

Transverse ricochetal brachiation is a movement strategy applied by climbing athletes when the target ledge is out of reach. In a single locomotion cycle, this gait can cover a longer distance and enables movement when there are no handholds. Figure 2a dissects the process of this gait. The athlete begins by swinging his lower limbs to accumulate energy. Once there is enough inertia, the athlete is able to reach out with his right hand. When this hand is about to touch the target ledge, he lets go with his left hand to release the accumulated energy completely. The duration of the flight phase in which neither hand is touching a ledge is extremely short (<0.1 s), so it is difficult to see this motion in the images captured in Figure 2a. Finally, the athlete grasps the target ledge with his right hand, and his left hand also swiftly resumes its position above the athlete’s head. This completes a single cycle of the transverse ricochetal brachiation gait. Based on the gait analysis results above, we divided this gait into the four phases shown in Figure 2b: swinging, approaching target, two-hand release, and hands-on.

Two common modes of locomotion can serve as a simple analogy to describe the two brachiation gaits above: walking and running. Walking is slower, but the entire process is steadier and saves energy. In contrast, running obviously has higher movement efficiency, but it consumes more energy during the process, and the posture of the body when both feet leave, and land on the ground influences the effectiveness and stability of this mode of locomotion. From the perspective of bionics, ricochetal brachiation can, to a certain extent, be compared to the trot gait of multiped robots. However, the gaits of brachiation robots must be paired with robust and coordinated ledge grip motions, as dissimilar to multiped robots, which can stand up and continue even after falling down [30], it is impossible for brachiation robots to return to a ledge once they fall. This is the greatest challenge in realizing continuous cycles in transverse brachiation.

### 2.3. Problem Statement

**Figure 3:**
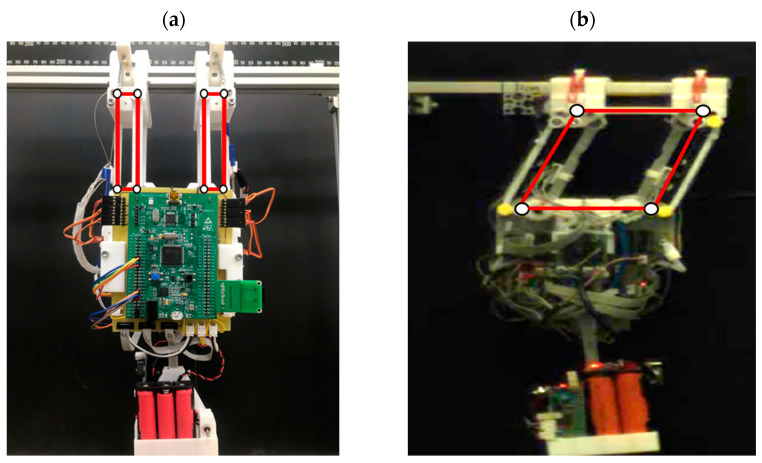
Mechanical design issues in existing transverse brachiation robots: (**a**) parallel links in dual arms; (**b**) parallel links constructed by two arms, horizontal ledge, and robot body.

Past studies [1,5] have highlighted two main challenges in the development of transverse brachiation robots. The first is that it is difficult to achieve two or more continuous cycles because brachiation robots generally rely on inertia to turn their bodies to facilitate ledge grasping [2]. However, this motion tends to create interference, which is a problem that must be considered carefully in mechatronic design. Furthermore, although the design in which parallel linkages are used in both arms [1] (as shown in Figure 3a) ingenuously keeps the robot’s body vertical to the ledge, it also reduces the freedom of body motions. If the body cannot smoothly rotate due to mechanism restrictions after the robot has grasped the ledge, the entire motion will be forced to stop. If the robot enters the hands-on phase in an awkward position, canceling out the energy accumulated during the swinging process. To delve deeper into this issue, we included the system dynamics of all of the phases in our robot gait analysis and redesigned suitable locomotion trajectories and the switching conditions to smoothly connect the various phases in accordance with the parallel four-link posture constraints (as shown in Figure 3b). The gait design objectives include enabling smooth transitions between movement phases, reducing the energy consumed to switch from one phase to the next, and maintaining the parallel four-link posture in the hands-on phase.

Another focus of this study was to enable the same robot configuration to realize a hybrid locomotion gait, including transverse brachiation and transverse ricochetal brachiation. This mechatronic realization problem has yet to be solved for ape-like brachiation robots. From Figure 1 and Figure 2, we can see that the main difference between the two types of brachiation lies in the third phase. With the transverse brachiation gait, both arms return to their original position in this phase for posture adjustment. Therefore, this phase is called the transition phase (Figure 1b). As for the transverse ricochetal brachiation gait, both grippers release at the same time during this stage for complete energy release, and inertia enables the fore gripper to grab the ledge. We defined this as the two-hand-release phase (Figure 2b). The other phases are similar; only the timing of switching between phases and the joint motion planning strategies differ slightly. This greatly increases the chance of successfully achieving these two types of gaits using the same robot configuration.

## 3. System Modeling

This study presents locomotion gait designs for transverse brachiation and transverse ricochetal brachiation with a locomotion cycle comprising four phases. The first two phases are the swinging phase and the approaching-target phase. The method of energy release in the third phase differentiates the two locomotion gaits. After both grippers have grasped the ledge, the robot adjusts its posture to help it enter the initial swing phase of the next locomotion cycle. The gait design contains a total of five different movement phases. We next give a brief introduction to the processes of establishing the dynamic model for each phase and the differences in the mathematical models of the phases. This serves as the foundation for gait parameter analysis and design.

We consider the example of a robot performing transverse brachiation to the right. Using the Euler–Lagrange method, we first define suitable origins for the coordinate system based on the various movement phases. Next, we select a state vector $q\in {\mathbb{R}}^{n\times 1}$ that can present the system dynamics completely. The external force input vector corresponding to the various states is $u\in {\mathbb{R}}^{n\times 1}$. With these settings, we can calculate the Lagrangian ℒ, including the kinetic energy T and potential energy V of the system, as follows:

(1) $$\begin{array}{l} \frac{d}{dt}\left(\frac{\partial ℒ}{\partial \dot{q}}\right)-\frac{\partial ℒ}{\partial q}=u\\ ℒ=T-V \end{array}$$

**Figure 4:**
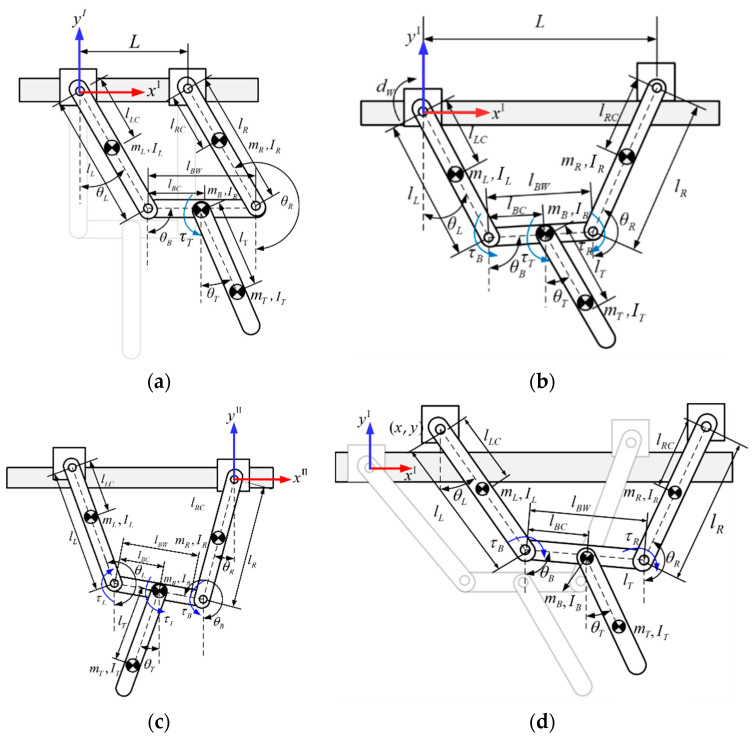
Schematic diagram of proposed transverse brachiation robot for classified phases Table 2. Robot model parameters. (**a**) Swing/Transition Phase. (**b**) Approaching-target Phase. (**c**) Hands-on Phase. (**d**) Two-Hand Release Phase. The red and blue arrows indicate the used coordinate system.

Based on the definition above, we can derive the dynamic equation of general standard robots as shown:

(2) $$M(q)\ddot{q}+C(q,\dot{q})+G(q)=u$$

where $M(q)\in {\mathbb{R}}^{n\times n}$ is the inertia matrix, $C(q,\dot{q})\in {\mathbb{R}}^{n\times 1}$ denotes the dynamic torques associated with the centripetal and Coriolis forces, and $G(q)\in {\mathbb{R}}^{n\times 1}$ represents the gravity torques. Figure 4 and Table 2, respectively, present the schematics of the proposed robot in each movement phase and the parameters associated with the system dynamic model. Next, we define the coordinate origin, system states, and input vectors of each phase to derive Equation (2) and explain the physical meaning and objective of the motions in each phase.

1. Swinging phase

In this phase, both grippers are gripping the ledge, and the arms form a parallel four-link geometric configuration with the environment. By swinging the tail, adequate energy is accumulated to facilitate the subsequent transverse movement. Based on the assumption of transverse locomotion to the right, we set the coordinate origin of this phase at the joint of the left wrist, *O_I_*, as shown in Figure 4a. Suppose the distance between the grippers in this phase is *L*, and the grippers are gripping the ledge firmly with no slippage occurring. Through the geometric relationships in the four-bar linkage mechanism [31], we can derive the relations of joint angles *θ_R_*, *θ_L_*, *θ_B_* as follows:

(3) $$\begin{array}{l} \theta_{R}(\theta_{L})=2\tan^{-1}(\frac{-k_{1}+\sqrt{k_{1}{}^{2}+k_{2}{}^{2}+k_{3}{}^{2}}}{k_{3}-k_{2}})\\ \theta_{B}(\theta_{R},\theta_{L})=\tan^{-1}(\frac{L-l_{L}\sin\theta_{L}+l_{R}\sin\theta_{R}}{l_{R}\cos\theta_{R}-l_{L}\cos\theta_{L}}) \end{array}$$

where $k_{1}=2l_{R}L-2l_{L}l_{R}\sin\theta_{L},\;k_{2}=-2l_{L}l_{R}\cos\theta_{L},\;k_{3}=L^{2}+l_{L}^{2}+l_{R}^{2}-l_{BW}^{2}-2l_{L}L\sin\theta_{L}$.

Equation (3) shows that right arm joint angle *θ_R_* and body joint angle *θ_B_* can be expressed as functions of left arm joint angle *θ_L_*. Thus, we can select the generalized coordinate vector $q_{\mathrm{swing}}={\left[\begin{array}{cc} \theta_{L} & \theta_{T} \end{array}\right]}^{T}$ and use two degrees of freedom to express the dynamics of the robot in this phase completely. This phase only uses the tail joint to perform system excitation, making it the equivalent of a two-link under-actuated system. Thus, we can let the input vector be $u_{\mathrm{swing}}={\left[\begin{array}{cc} 0 & \tau_{T} \end{array}\right]}^{T}$.

2. Approaching-target phase

After the tail has accumulated enough energy by swinging back and forth, the robot can switch to this phase at an appropriate time and reach for the target ledge; that is, the right hand can let go of the ledge and grasp the ledge to the right as shown in Figure 4b. In this phase, the robot’s left gripper still has a firm grip on the ledge, and assuming there is no slipping between the gripper and the ledge, the origin of the coordinate system is set at the left wrist joint, *O_I_*, as exhibited in Figure 4b. The robot’s right gripper has let go in this phase, so the right arm is not subject to the geometric constraints as it was in the previous phase. Thus, we select a generalized coordinate vector $q_{\mathrm{target}}={\left[\begin{array}{cccc} \theta_{L} & \theta_{B} & \theta_{R} & \theta_{T} \end{array}\right]}^{T}$. In this phase, the left wrist has no actuation torque. During the movement, the shoulder joints and the tail joint provide the actuation torque ($\tau_{B},\tau_{R},\tau_{T}$), which, along with the energy accumulated in the swing phase, is used to reach for and grasp the target ledge. Thus, the input vector is defined as $u_{\mathrm{target}}={\left[\begin{array}{cccc} 0 & \tau_{B} & \tau_{R} & \tau_{T} \end{array}\right]}^{T}$.

3. Transition phase

After the robot has successfully grasped the ledge in the previous approaching-target phase, it returns to having both grippers gripping the ledge at the same time. Here, we designed a posture transition phase to help the robot return smoothly to its original posture at the beginning of a gait cycle. During this stage, the upper limbs similarly form a four-link configuration with the ledge as they perform in the swing phase, and the robot relies on swinging its tail to correct its overall posture and help the other gripper move forward to its original position. As shown in Figure 4a, the coordinate system, generalized coordinates, and input vectors are identical to those in the swing phase, which means that the mathematical dynamic model is the same. However, essential differences exist between the joint motion trajectories and planning objectives of these two phases.

4. Hands-on phase

Once the robot adjusts to a certain posture in the previous phase, the left gripper releases the ledge and moves to the right back to its original position so that the distance between the two grippers returns to the set distance in the initial swing phase, the posture of the body and the tail is coordinated, and the robot can again begin the excitation and energy storage phase needed for the next locomotion cycle. This can be regarded as a successful locomotion gait cycle. In this phase, we assume that the left gripper releases the ledge as the right gripper has a firm grip on the ledge with no slippage. Thus, the coordinate origin is set at the right wrist joint *O_II_*, as shown in Figure 4c. The generalized coordinate variable of gripping with one gripper is identical to that in the target-approaching phase, that is, $q_{\mathrm{hbo}}={\left[\begin{array}{cccc} \theta_{L} & \theta_{B} & \theta_{R} & \theta_{T} \end{array}\right]}^{T}$ and the external force input vector is set $u_{\mathrm{hbo}}=\left[\begin{array}{cccc} \tau_{L} & \tau_{B} & 0 & \tau_{T} \end{array}\right]$ based on the actuation joint torque.

Reminder: The system state variable $q_{\mathrm{hbo}}$ in this phase is defined based on the right wrist joint coordinate system (*O_II_*) (Figure 4c) and is different from the status variable $q_{\mathrm{target}}$ in the second phase, which was defined based on the left wrist joint coordinate system (*O_I_*) (Figure 4b). The conversion formulas of the joint angles with regard to these two coordinate systems are as follows:

(4) $$\left\{ \begin{array}{l} \theta_{L}^{\mathrm{II}}=\theta_{L}^{I}+\pi \\ \theta_{B}^{\mathrm{II}}=\theta_{B}^{I}+\pi \\ \theta_{R}^{\mathrm{II}}=\theta_{R}^{I}-\pi \\ \theta_{T}^{\mathrm{II}}=\theta_{T}^{I} \end{array}\right.$$

5. Two-hand-release phase

In this study, the four movement phases above can be used to express the entire dynamics of the transverse brachiation locomotion gait. However, ricochetal brachiation requires a description of the dynamics when both grippers have released the ledge, and the robot is airborne. We use the two-hand-release phase in Figure 4d to represent this. In ricochetal brachiation to the right, we chose to set the coordinate origin at the left wrist joint *O_I_*. While the robot is airborne, there are no geometric constraints for its joints, and there is additional two-dimensional translation along the *xy* plane. Thus, the state variable that we chose for this phase was $q_{\mathrm{thr}}={\left[\begin{array}{c} \begin{array}{c} \begin{array}{cccccc} \theta_{L} & \theta_{B} & \theta_{R} & \theta_{T} & x & y \end{array} \end{array} \end{array}\right]}^{T}$. The goal of this phase is to use the actuators at the shoulder joints $\left(\tau_{B},\tau_{R}\right)$ to enable the two shoulders of the robot to move toward the target ledge during the extremely short leap. During this process, the tail actuator $\left(\tau_{T}\right)$ maintains a certain posture (i.e., a fixed tail joint angle) to reduce its impact on the swinging motions of both shoulders during flight and thereby increase the landing success rates of both grippers. The external force input vector is defined as $u_{\mathrm{thr}}={\left[\begin{array}{c} \begin{array}{c} \begin{array}{cccccc} 0 & \tau_{B} & \tau_{R} & \tau_{T} & 0 & 0 \end{array} \end{array} \end{array}\right]}^{T}$.

Based on the coordinate system, state variable, and input vector defined above, we can derive the system dynamic equation of each phase (Equation (2)), where Phases 1-2-3-4 represent a transverse brachiation locomotion cycle and Phases 1-2-5-4 represent a transverse ricochetal brachiation locomotion cycle. In the next section, we analyze the design parameters that meet the dual-arm robot configuration conditions to enable smooth transitions.

## 4. Robot Locomotion Design

Based on the derived dynamic model, this section analyzes the design goals needed to complete an entire locomotion cycle. On the premise of the causal relationship, we explain the means of selecting switch parameters to achieve smooth transitions between phases. We then propose corresponding motion control strategies. We referred to the modified version of a transverse brachiation robot prototype already developed by our lab [1,2] for the required design parameters.

### 4.1. Locomotion for Transverse Brachiation

To achieve the goal of continuous transverse locomotion, we set two design goals for the robot locomotion gait:

(1) each movement phase must be executed successfully, and two continuous locomotion gait cycles must be completed; in other words, eight movement phases must be completed continuously;
(2) the energy connecting the motion processes of each phase must be maintained and not return to zero at the end of which phase, which would cause uncoordinated motions.

**Figure 5:**
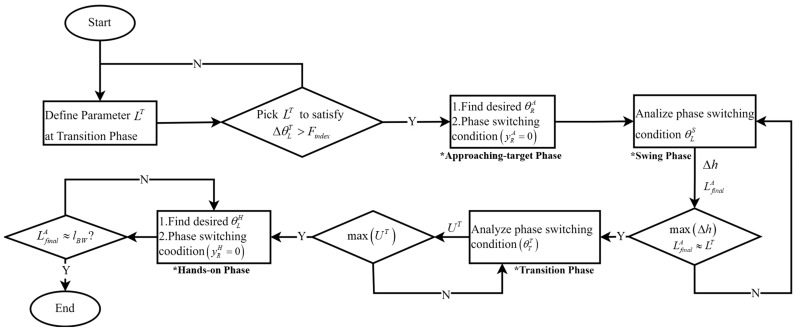
Flow chart of locomotion control design strategy for transverse brachiation. * use to highlight each different phase.

The proposed process for the design of transverse brachiation locomotion gaits is presented in the flow chart in Figure 5. For the sake of convenience, we use the following abbreviations for each phase in the parameters and explanation: swinging phase (S), approaching-target phase (A), transition phase (T), and hands-on phase (H). As seen in the figure, this design method does not analyze the switching conditions in the S→A→T→H phase order but first defines a flexibility index first to obtain the maximum relative distance between the two grippers (the reason for which is explained later). Once the maximum gripper distance and upper limb assumptions have been obtained, mechanical geometric relationships are used to derive the arm joint motion trajectories for the approaching-target phase and the switching conditions for posture adjustment. The next step is to analyze the swinging phase parameters while meeting the aforementioned assumptions to obtain the appropriate timing to initiate reaching and gripping. The dynamic simulation results obtained from the switching conditions of the first two phases are substituted into the dynamic model of the posture transition phase. This facilitates the selection of the operating parameters needed to switch to the next phase. Finally, the posture condition parameters of the motions completed in the previous phase are substituted into the dynamic model of the hands-on phase to derive the position where the left arm needs to go so that it is in the same position as it was in the initial cycle, as well as the switching conditions for the swinging phase of the next cycle. In the next section, we explain the logic of this design process in detail and the reasons underlying the selection of the switching conditions.

#### 4.1.1. Design Requirements and Analysis Procedure for Transverse Brachiation

**Figure 6:**
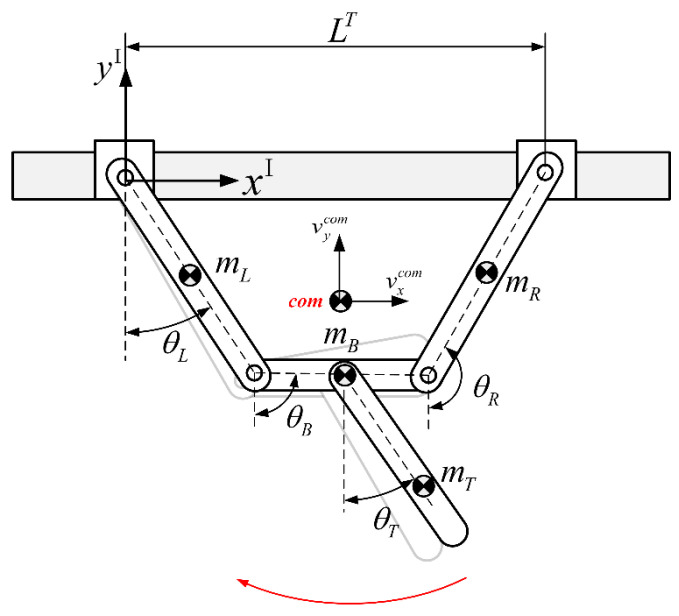
Schematic diagram of transition phase where design parameter *L^T^* is related to the range of motion of upper limb (Δ*θ_L_*). Red arrow indicates moving direction.

In the gait analysis in Section 2, we observed that the athlete did not grasp the furthest position in the approaching-target phase but maintained a suitable distance between his hands. This creates the necessary posture for the next phase. Thus, we first analyzed the influence of the distance between the two hands in the transition phase on the entire locomotion gait. As shown in Figure 6, we first defined the distance between the two grippers after the robot switches from the approaching-target phase to the posture transition phase as the design parameter $L^{T}$. Note that a greater distance is not superior; an overly-far grasping distance increases the rigidity of the entire robot and reduces the range at which the robot’s body can rotate as the tail swings for posture adjustment. In the worst-case scenario, the body will be completely unable to adjust its posture, thereby forcing the entire locomotion gait to stop.

**Figure 7:**
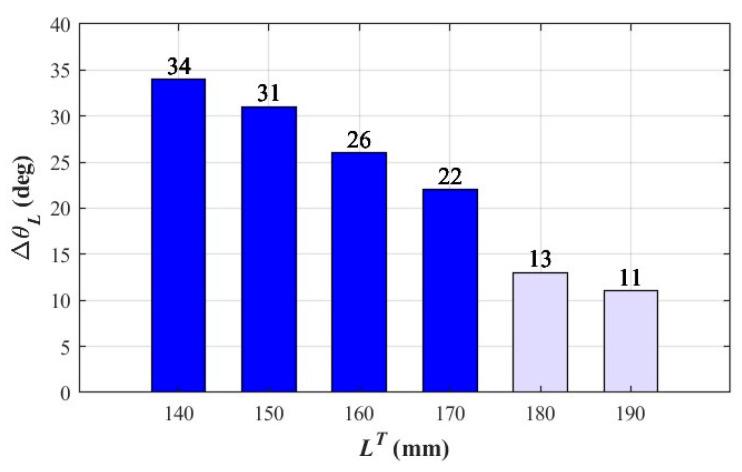
Relationship between yaw angle (Δ*θ_L_*) and *L^T^*. Deep blue bars indicate the results substantially distinguished from others.

We assumed that the upper limbs for an isosceles trapezoid with the ledge at the beginning of the transition phase; that is $\theta_{B}=90{}^{\circ}$ in Figure 6. If we issue a swinging command to the lower limb, we can see a clear connection between the $L^{T}$ distance between the two grippers and $\Delta \theta_{L}$ the yaw angle of the arms, which is defined as

(5) $$\Delta \theta_{L}≜\left|\theta_{L\_\max}^{T}-\theta_{L\_\min}^{T}\right|\geq \;F_{index}$$

where $\theta_{L\_\max}^{T}$ and $\theta_{L\_\min}^{T}$ are the maximum and minimum values of $\theta_{L}$ the angle of the left arm as the tail is swinging in the transition phase. Thus, the difference $\Delta \theta_{L}$ is the permissible swing range of the left arm. The larger this range is, the more flexible the motions of the upper limbs are and the less likely it is for dead-point positions or locked chains in this phase. In Equation (5), we define the flexibility index $F_{index}$ to represent the range in which the upper limbs of the robot can move with different distances between the two grippers. When the upper limbs and the ledge form a parallel four-link posture (i.e., the initial state of the swing phase), the robot is the most flexible; however, if the relative distance between the grippers increases after the robot enters the transition phase, the upper limbs will become less flexible. To increase the grasping distance as much as possible while maintaining a certain range in which the arms can move to facilitate smooth transitions to subsequent movement phases, we analyzed several different $L^{T}$ values, and after performing mechatronic system tests, we set *F_index_* at 20°. As shown in Figure 7, the dark blue bars represent results that meet the flexibility index, whereas the light purple bars represent results that do not meet the flexibility index. As can be seen, when $L^{T}$ is equal to 170 mm, the range in which the arms can move is 22°, which is not too different from the 26° that the arms can move when $L^{T}$ is equal to 160 mm. However, if the distance between the two grippers increases to 180 mm, the range in which the arms can move drops significantly to 13°. Thus, the design parameters obtained using *F_index_* = 20° represent a good compromise. Following the selection of this parameter value, we introduce the switching conditions and motion planning strategies of the approaching-target phase, the swinging phase, the transition phase, and the hands-on phase according to the steps shown in Figure 5.

#### 4.1.2. Motion Control Strategy Associated with Approaching-Target Phase for Transverse Brachiation

The analysis in the previous section shows that at the beginning of the transition phase, the desired distance between the two grippers, $L^{T}$, is 170 mm, and the upper limbs form an isosceles trapezoid. To meet these conditions, we planned the joint motion trajectories of the robot in the previous movement phase as follows:

(6) $$\left\{ \begin{array}{l} \theta_{R}^{A}(t)=180^{{}^{\circ}}-\tan^{-1}\frac{\sin\theta_{L}^{A}(t_{f})}{\sqrt{1-\sin^{2}\theta_{L}^{A}(t_{f})}}\\ \tau_{L}^{A}(t)=0\\ \theta_{T}^{A}(t)=\theta_{T}^{S}(t_{f})\\ \theta_{B}^{A}(t)=90^{{}^{\circ}}\\ \sin\theta_{L}^{A}(t_{f})=\frac{L^{T}-L_{BW}}{2l_{L}} \end{array}\right.$$

In the approaching-target phase, the transmission of motor torque can be stopped by switching the clutch on the left wrist. Thus, $\tau_{L}^{A}(t)=0$, which allows the four-link robot to freely rotate its left wrist and coordinate with the other three actuated joints to achieve the expected grip posture using proportional-derivative (PD) control. During the grasping process, we had the tail joint angle remain the same as it was at the end of the swing phase ($t_{f}$); in other words, $\theta_{T}^{A}(t)=\theta_{T}^{S}(t_{f})$. With the isosceles trapezoid restriction, the conditions of the body joint angle $\theta_{B}$ and right arm joint angle $\theta_{R}$ are $\theta_{B}^{A}(t_{f})=90^{{}^{\circ}}$ and $\theta_{R}^{A}(t_{f})+\theta_{L}^{A}(t_{f})=180^{{}^{\circ}}$, where $\theta_{L}$ is the feedback value of the left arm joint angle obtained in the event of no torque input. Based on this geometric restriction, we can obtain $\theta_{R}^{A}(t)$, the reference command of $\theta_{R}$ given during the grasping process, as shown in Equation (6). After the right gripper has grasped the ledge (i.e., the coordinate value $y_{R}^{A}=0$ in the vertical direction of the right gripper in Figure 4b), the robot can switch to the next phase (i.e., transition). The switching condition can be expressed as

(7) $$y_{R}^{A}=-l_{L}\cos\theta_{L}-l_{\mathrm{BW}}\cos\theta_{B}-l_{R}\cos\theta_{R}=0$$

#### 4.1.3. Motion Control Strategy Associated with Swinging Phase for Transverse Brachiation

**Figure 8:**
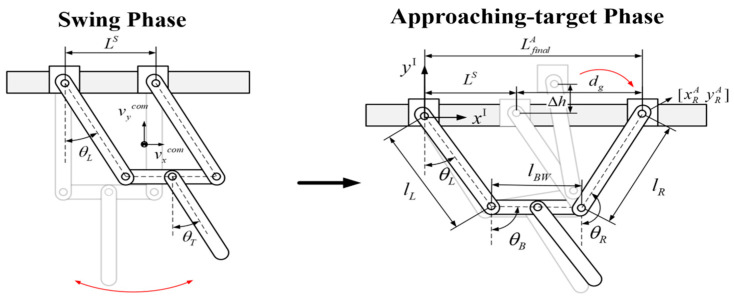
Schematic diagram of transition from swinging to approaching-target phases: robot releases its right hand to approach the target position as the swung arm satisfies phase switching condition $\theta_{L}=28.8^{{}^{\circ}}$.

Based on the aforementioned assumption and switching condition in Equation (7), we analyzed the parameters of the swinging phase and the conditions needed for motion planning. We sought to determine the appropriate timing to switch from the swinging phase to the approaching-target phase. Figure 8 presents a schematic diagram of the transition between the two phases. As mentioned previously, the goal of the swing phase is to swing the lower limb back and forth while the two arms are in a parallel ledge-gripping posture to store enough system kinetic energy to reach the target grasping distance. At this point, the distance between the two grippers is $L^{S}=L_{BW}$. By switching the clutches in both wrists, there is no torque input in both arm joints so they can rotate freely. The reference trajectory given to the tail joint motor is a sine function, and closed-loop system control is realized using the PD control law. Due to mechanical restrictions, we set the maximum amplitude of the sine function to 45°. Using a swept sine, we can derive that the resonant frequency of the robot system in the swing phase is approximately 1.5 Hz. The simulation results show that in the fourth swing, the system will enter a resonant state. Thus, three swings were performed before the conditions derived later were used to switch to the target-approaching phase during the fourth swing.

As shown in Figure 8, a greater height difference (Δh) between the highest point of the right gripper after releasing the ledge and the height of the target ledge means a longer duration of flight as well as more flexibility in the designs of joint motion planning. As the direction of the speed is the same as the direction in which the system is moving, the y-direction speed component of the centroid at the instance of transition from the swing phase to the target-approaching phase should be greater than 0 ($v_{y}^{com}\;>\;0$), and the direction of the angular velocity of the left arm joint should be counterclockwise (${\dot{\theta }}_{L}\;>\;0$). This enables the right arm to leave the ledge the right gripper was originally gripping and reach the right to grasp the target ledge successfully. With the target distance between the two grippers ($L_{\mathrm{Target}}^{A}$) in the target-approaching phase meeting the previously set parameter $L^{T}=170\;\mathrm{mm}$ minus the initial distance between the two grippers ($L^{S}=100\;\mathrm{mm}$), the target moving distance of the arms ($d_{g}$) is approximately 70 mm. We, therefore, chose the arm joint angle $\theta_{L}$ during the swing phase as the basis for the choice of when to switch to the target-approaching phase. Based on the aforementioned constraints, the height of the right gripper in the flight phase after it releases the ledge, must be as high as possible. We can therefore categorize the following optimization problems:

(8) $$\begin{array}{l} \underset{\theta_{L}}{\arg}\;\max\{\Delta h\;(\theta_{L},v_{y}^{com},\;{\dot{\theta }}_{L})\}\;\\ \mathrm{subject}\;\mathrm{to}\;v_{y}^{com}\;>\;0\;,\;{\dot{\theta }}_{L}>0,\;L_{\mathrm{Target}}^{A}\approx 170\;(\mathrm{mm})\; \end{array}$$

**Figure 9:**
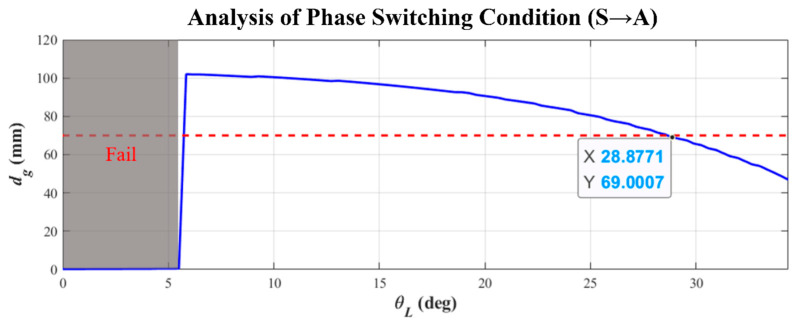
Relationship between phase switching (S→A) condition *θ_L_* and gripper grasping distance *d_g_*.

Using dynamic simulation analysis, we derived that the phase switching condition (S→A) satisfying Equation (8) is for the right gripper to release the ledge to enter the approaching-target phase when the arms swing to $\theta_{L}=28.8^{{}^{\circ}}$ in the swinging phase. Figure 9 presents the relationship between the switching condition ($\theta_{L}$) and the grasping distance ($d_{g}$) that can be completed in the target-approaching phase. The results where phase switching cannot be achieved are in the gray area. As can be seen, $\theta_{L}$ must be greater than about 6° in order for the robot’s right gripper to leave the swinging phase successfully. The red horizontal dashed line represents the target movement distance for the right gripper (70 mm). We relaxed this constraint slightly to 69 mm (i.e., $L_{\mathrm{final}}^{A}=169\;\mathrm{mm}$) so that more energy can be accumulated during the swinging phase to compensate for any energy consumption that the dynamic model does not take into account. Thus, the corresponding phase switching condition is $\theta_{L}=28.8^{{}^{\circ}}$.

#### 4.1.4. Motion Control Strategy Associated with Transition Phase for Transverse Brachiation

As shown in Figure 6, once the right gripper grasps the ledge, the goal of the posture transition phases is added, in which the posture of the upper limbs is adjusted by swinging the tail, and the potential energy of the entire system is also increased to help the left arm return to its original position. To this end, we used the same sine function as that used in the swinging phase as reference input of the tail joint angle ($\theta_{T}^{T}(t)$). Increasing the potential energy U of the entire system was the primary goal, and the tail swing angle $\theta_{T}$ was chosen as the switching parameter determining when to transition to the next phase (T→H). U can be derived using the following equation:

(9) $$U=m_{L}g{\vec{p}}_{L}^{y}+m_{B}g{\vec{p}}_{B}^{y}+m_{R}g{\vec{p}}_{R}^{y}+m_{T}g{\vec{p}}_{T}^{y}$$

where g is the gravitational acceleration constant and ${\vec{p}}_{i}^{y}\left(i=L,B,R,T\right)$ is the vertical component of the position vectors from the origin of the coordinate system in this phase to the centroids of each link. Similarly, the *y* component of the system centroid speed at the instance of phase switching should also be greater than 0 ($v_{y}^{com}\;>\;0$) to prevent the robot from falling when it releases the ledge. Furthermore, the direction of the angular velocity of the left arm joint should be clockwise (${\dot{\theta }}_{L}<0$) to coordinate with the movement of the left arm to the right to return to its original position.

**Figure 10:**
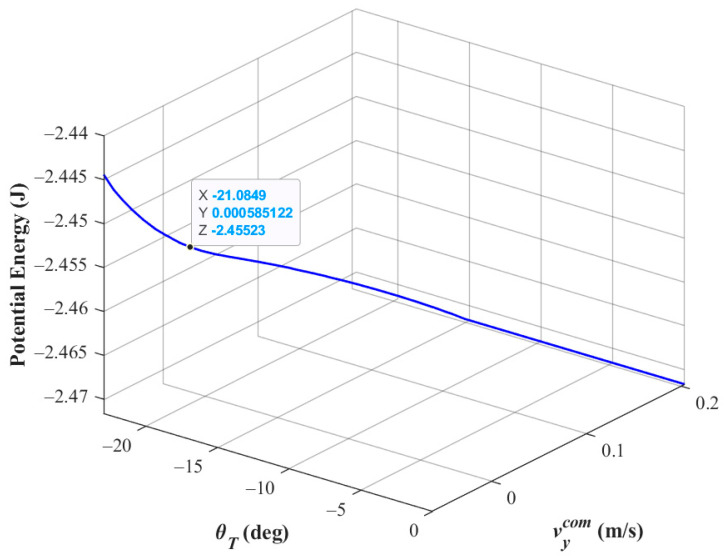
Parameter analysis of phase switching condition from the transition phase to the hands-on phase where the robot shifts to the next phase when the joint angle of the tail link swinging from right to left is $\theta_{T}=-21.08^{{}^{\circ}}$.

Combining the above conditions, we can establish an optimization problem as follows:

(10) $$\begin{array}{l} \underset{\theta_{T}}{\arg}\;\max\{U^{T}(\theta_{T},v_{y}^{com},\;{\dot{\theta }}_{L}\;)\}\;\\ \mathrm{subject}\;\mathrm{to}\;v_{y}^{com}\;>\;0\;,\;{\dot{\theta }}_{L}<0\; \end{array}$$

where $U^{T}$ denotes the potential energy of the system in the transition phase. Figure 10 shows the parameter analysis results derived from the computer simulation; the X, Y, and Z axes, respectively, represent the phase-switching condition parameter ($\theta_{T}$), the y component of the system centroid speed ($v_{y}^{com}$), and the potential energy of the system (*U*). From the figure, we can see that the critical value of potential energy at which the robot can maintain an upward overall centroid speed is approximately −2.455 J. At this point, the lower limb swinging to $\theta_{T}=-21.08^{{}^{\circ}}$ (a negative value indicates the clockwise direction) is the appropriate time to switch to the next movement phase.

#### 4.1.5. Motion Control Strategy Associated with Hands-on Phase for Transverse Brachiation

**Figure 11:**
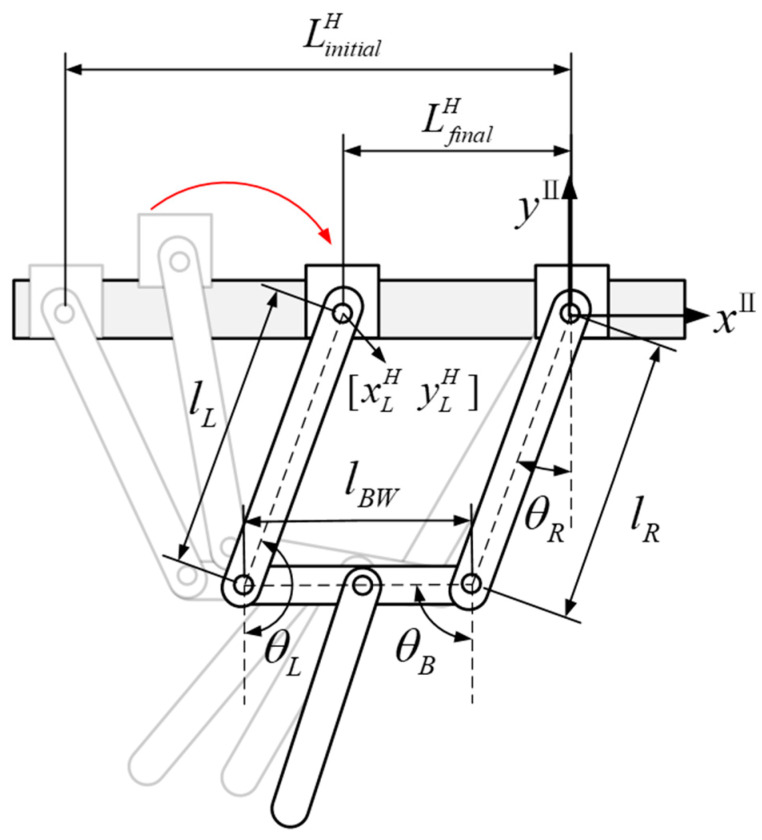
Schematic diagram of the hands-on phase where grey indicates the motion process and black indicates the robot has returned to the initial posture (i.e., swinging phase).

Following the analysis of the first three phases, we substitute the known operating parameters into the dynamic model of the hands-on phase for analysis to complete the gait design. The motions of this phase are shown in Figure 11. The primary design goals of this phase are (1) enabling the robot to complete this transverse locomotion cycle successfully and (2) making the geometric structure of the upper limbs after the left gripper grasps the ledge as close to a parallel four-link posture (i.e., the distance between the two grippers $L_{final}^{H}\approx l_{BW}=100\;\mathrm{mm}$) as possible so that the robot can again perform the next locomotion cycle and achieve continuous brachiation. Thus, the various joints must meet the following conditions:

(11) $$\left\{ \begin{array}{l} \tau_{R}^{H}(t)=0\\ \theta_{T}^{H}(t)=\theta_{T}^{T}(t_{f})\\ \theta_{L}^{H}(t)=\theta_{L}^{H}(t_{f})\\ \theta_{B}^{H}(t)=-90^{{}^{\circ}}\\ \theta_{L}^{H}(t_{f})-\theta_{R}^{H}(t_{f})=180^{{}^{\circ}} \end{array}\right.$$

In this phase, the right wrist joint can rotate freely (by switching the clutch); therefore, $\tau_{R}^{H}(t)=0$. The tail joint angle command ($\theta_{T}^{H}(t)$) is set at the angle it was at the end of the previous phase. $\theta_{B}^{H}(t_{f})=-90^{{}^{\circ}}$ and $\theta_{L}^{H}(t_{f})-\theta_{R}^{H}(t_{f})=180^{{}^{\circ}}$ are the joint position command conditions needed for the body joint angle $\theta_{B}$ and left arm joint angle $\theta_{L}$ to form the parallel four-link posture at the end of this phase ($t_{f}$), whereas the right arm joint angle $\theta_{R}$ equals the feedback value transmitted back via the encoder where there is no torque input. Thus, the angle command given to the left arm joint $\theta_{L}$ is $\theta_{L}^{H}(t_{f})$. Note that the coordinate origin in this phase is located at the wrist joint of the right gripper. The positive and negative joint angles in this phase differ from those in the other phases (counterclockwise direction defined as positive). Furthermore, the initial distance between the two grippers in this phase is $L_{initial}^{H}=L^{T}$.

**Figure 12:**
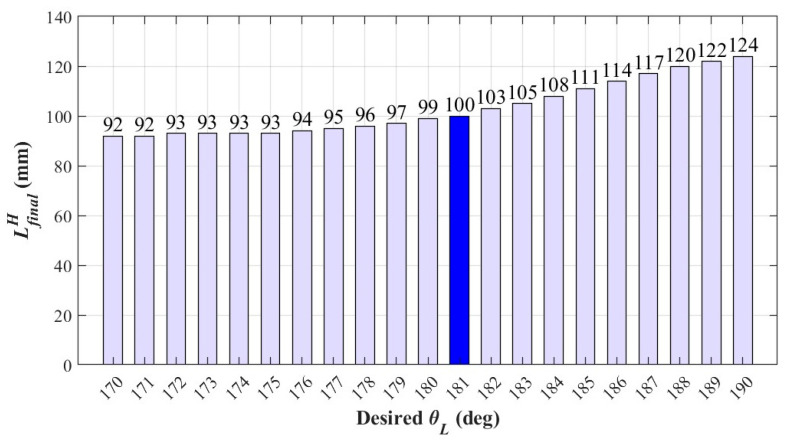
Parameter analysis of the hands-on phase where the upper limb posture represents a parallelogram if the target value of the left arm joint angle $\theta_{L}(t_{f})$ is 181° (the one with deep blue bar).

To achieve the posture in Equation (11), performing simulation analysis using the dynamic model of this phase produces the relationship between the final value of the left arm joint angle $\theta_{L}(t_{f})$ and the distance between the two grippers after gripping, as shown in Figure 12. As can be seen from this figure, setting $\theta_{L}(t_{f})=181{}^{\circ}$ results in $L_{final}^{H}=L_{BW}=100\;\mathrm{mm}$. This means that after the left gripper has grasped the ledge again, the geometric posture of the upper limbs is closest to a parallel four-link posture, as shown in Figure 11. When the y coordinate of the wrist of the left gripper is 0 ($y_{L}^{H}=0$), the robot can return to the swinging phase to store energy for the next locomotion cycle; the phase switching (H→S) condition is

(12) $$y_{L}^{H}=-l_{R}\cos\theta_{R}-l_{BW}\cos\theta_{B}-l_{L}\cos\theta_{L}=0$$

Following a comparison with Figure 5, we find the phase switching conditions to achieve continuous transverse brachiation locomotion can be re-categorized as follows:

1. Swinging phase→Approaching-target phase: arm swing angle becomes $\theta_{L}^{S}=28.8^{\circ }$;
2. Approaching-target phase→Transition phase: vertical position of grasping gripper coincides with ledge position $y_{R}^{A}=0$;
3. Transition phase→Hands-on phase: tail link swings back until joint angle $\theta_{T}^{T}=-21.1^{\circ }$;
4. Hands-on phase→Swing phase: vertical position of returning gripper coincides with the ledge position $y_{L}^{H}=\;0$.

### 4.2. Locomotion for Transverse Ricochetal Brachiation

The design proposed in Section 4.1 is based on postures with at least one hand holding onto the ledge. The energy accumulated during the swinging phase is lost to a certain degree under the constraints, so the grasping distance must allow for posture adjustment and mechanical flexibility when both grippers are gripping the ledge. For this reason, we propose incorporating a leap motion during the ledge gripping process. A leaping motion would more effectively utilize the energy accumulated during the swinging phase and increase the gait distance and mobility performance of the robot. In other words, we propose replacing the posture adjustment phase of the TLB gait with a two-hand-release phase to design a TLRB gait. The timing of switching from the approaching-target phase to both hands letting go for the flight phase is crucial. The wrong switching conditions will affect the subsequent landing posture, the grasping distance, and the success of the arms returning to their original position after landing.

**Figure 13:**
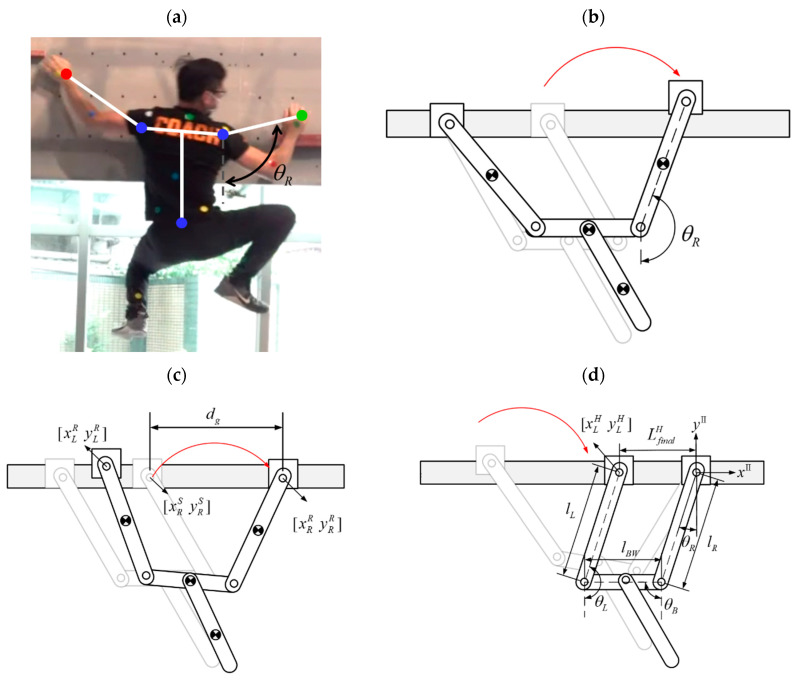
Schematic diagram of TLRB locomotion design: (**a**) athlete posture in approaching-target phase; (**b**) robot posture in approaching-target phase; (**c**) grasping distance *d_g_*; (**d**) robot posture after finishing hands-on phase. Red arrow indicates moving direction.

**Figure 14:**
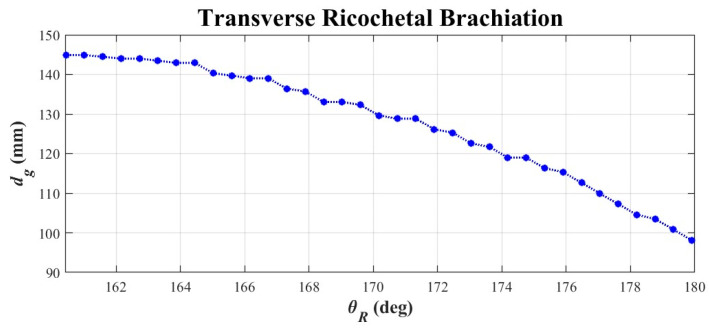
Analysis of phase switching condition *θ_R_* and grasping distance *d_g_* for TLRB (timing for the robot to release ledge-holding hand is related to posture of approaching-target arm).

Figure 13 presents a schematic diagram of the motions in the TLRB gait after the fore gripper has grasped the ledge. We applied the trajectory planning results of TLB as the basis of the gait design under the following design process:

1. The first step in the two-hand-release phase of TLRB is realizing the leap. However, as the left gripper releases the ledge in the instant before the right gripper reaches the ledge, we can assume that the arms begin to resume their original position once the right gripper has grasped the ledge. Based on the joint trajectory planning represented by Equation (11) for the TLB gait, the upper limbs almost form a parallelogram once the left arm resumes its original position. This can be referred to as the locomotion cycle of a TLRB gait.
2. As the execution of the flight stage is short, the various joint angle commands of the robot remain the same as they were at the end of the previous phase (i.e., the joint angles at the instant the left gripper releases the ledge).
3. We adopted the same parameter settings as those of the TLB gait for the initial robot posture, excitation frequency, and amplitude in tail joint trajectory planning.
4. Unlike the TLB gait, where the need for a posture adjustment phase causes the grasping distance to be restricted by mechanical flexibility, this gait mainly uses the energy accumulated in the swinging phase to lengthen the grasping distance as much as possible. The analysis results in Figure 9 show that as the TLRB gait does not require the posture adjustment phase, the constraint $L_{\mathrm{Target}}^{A}=170\;\mathrm{mm}$ can be removed from Equation (8). In this way, the grasping distance $d_{g}$ in the approaching-target phase is no longer restricted by mechanical flexibility. In other words, when the angle of the left arm in the swinging phase is $\theta_{L}=5.84^{\circ }$, the right gripper can release the ledge, and the inertia of the robot can be effectively utilized to achieve the maximum grasping distance $d_{g}=101\;\mathrm{mm}$. Thus, we chose $\theta_{L}=5.84^{\circ }$ as the design condition for switching from the swinging phase to the approaching-target phase. Note that these are only the results derived based on the analysis of the dynamic models in the first two phases of the TLB gait. Later, the energy accumulated in previous phases can be further used to increase the total grasping distance during the leap.
5. As shown in Figure 13c, the robot switches to the hands-on phase once its fore (right) gripper grasps the ledge in the two-hand-release phase. The switching condition can be expressed using the following equation:
  (13) $$y_{R}^{R}=-l_{R}\cos\theta_{R}-l_{BW}\cos\theta_{B}-l_{L}\cos\theta_{L}+y_{L}^{R}=0$$
  where $y_{R}^{R}\;\mathrm{and}\;y_{L}^{R}$ are the *y* coordinates of the left and right gripper joints in the two-hand-release phase.
6. As shown in Figure 13d, the hands-on phase mainly utilizes the horizontal inertia the robot obtained to leap. After the right gripper grasps the ledge, the left arm joint uses Equation (11) to bring the left gripper forward to grasp the ledge, thereby completing a TLRB locomotion cycle. At this point, the upper limbs of the robot again for a parallel four-bar linkage and the y coordinate of the left gripper joint $y_{L}^{H}=0$ can also serve as the basis of judgment for the swing phase in the next locomotion cycle.
  (14) $$y_{L}^{H}=-l_{R}\cos\theta_{R}-l_{BW}\cos\theta_{B}-l_{L}\cos\theta_{L}=0$$
7. Figure 13a,b exhibit the schematics of the TLRB gait in the target-approaching phase. The focus of this movement phase is the timing of when the left gripper releases the ledge to perform the leap motion after the right gripper reaches out for a good grasping distance ($d_{g}$). Here, we used the right arm joint angle ($\theta_{R}$) as the basis for phase switching, as shown in Figure 13c. Note that the $d_{g}$ here is based on the ideal grasping distance derived from the dynamic analyses of the approaching-target phase and the flight phase and is different from the results obtained from the analysis in the previous section, which did not consider flight dynamics. Based on the above assumptions, we conducted a dynamic simulation of the entire gait using the switching conditions of the previous phases. As can be seen in Figure 14, there is a greater $\theta_{R}$ increase in the amount of time that the right gripper is airborne after the left gripper releases the ledge, and this increases the landing success rate. However, the grasping distance also decreases as the height of the right arm increases. Considering the mechanical restrictions of the body and arm links in the robot hardware, we chose to have the robot’s left gripper release the ledge for the leap motion when the right arm raises to $\theta_{R}=170^{\circ }$ in the approaching-target phase. The resulting grasping distance is approximately 130 mm. Note that only when the robot is not airborne in the instant the right gripper releases the ledge is phase-switching considered successful. Simulations with this condition parameter revealed that $L_{final}^{H}$ the distance between the two grippers in the hands-on phase is around 127 mm. To move closer to the upper limb ledge grip posture of parallel four-bar linkage, a smaller $\theta_{R}$ reference value can be used as the phase-switching condition. However, this may also cause the flight duration of the right arm to be too short and result in gait failure during mechatronic system realization.

The phase-switching conditions of the transverse brachiation locomotion gait proposed in this study can be organized as follows:

1. Swinging phase→Approaching-target phase: arm swing angle becomes $\theta_{L}^{S}=5.8^{\circ }$;
2. Approaching-target phase→Two-hand-release phase: robot releases the ledge-holding (left) hand when the joint angle of the approaching (right) arm reaches $\theta_{R}^{A}=170^{\circ }$;
3. Two-hand-release phase→Hands-on phase: vertical position of approaching hand coincides with ledge position $y_{R}^{R}=0$;
4. Hands-on phase→End: vertical position of returning hand coincides with the ledge position $y_{L}^{H}=\;0$.

## 5. Experiments and Discussion

In this section, we apply joint trajectory planning and phase-switching conditions derived from the previous analysis to a self-made transverse brachiation robot. We performed experiments with the two types of locomotion gaits to verify the feasibility of the proposed design and evaluate its locomotion performance.

### 5.1. Experiment Setup

**Figure 15:**
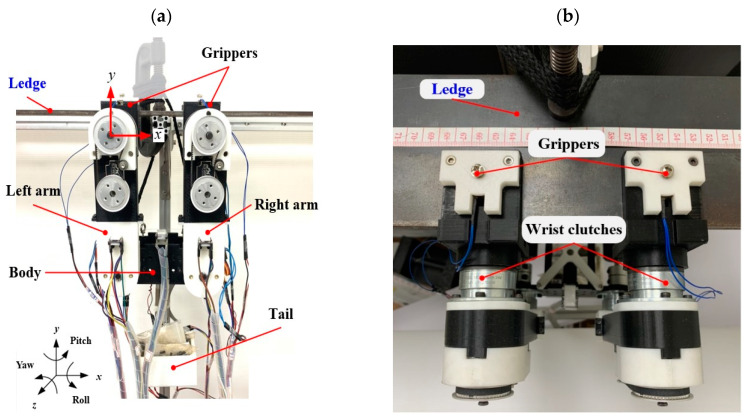
Test scenario for transverse brachiation experiments: (**a**) front view; (**b**) top view.

Figure 15 shows a transverse ledge climbing scenario consisting of an aluminum extrusion and a long metal plate. Figure 15a shows the front view of the initial state of the robot grasping the ledge, and Figure 15b presents the top view. The grippers have a one-piece mechanical design similar to “Γ” in shape and use solenoid electromagnets to switch between gripping and releasing swiftly. We installed electromagnetic clutches between the shoulder joints and wrist joints of the robot to switch to the under-actuated mode for system excitation in the swinging phase. In the gripping and hands-on phases, it is switched to the servo mode to control gripper-end posture using belt transmission.

This study adopted a four-link robot consisting of two arms, a body, and a tail to realize anthropomorphic TLB. The shoulder and lower limb joints of the robot are equipped with DC motors with encoders, and we employed the STM32F407 Discovery board to realize the algorithms needed for joint motion control and the overall locomotion gait. Inertial measurement units are installed on the arm and body links, and the motor encoders transmit the various joint angle measurement results wirelessly to a personal computer via a Bluetooth module for further analysis. For a detailed description of the system architecture, please refer to [2].

### 5.2. Experiments for Transverse Brachiation

We first present experimental gait results for a single locomotion cycle and then add experimental results for two locomotion cycles to explain the possible problems and research challenges in the realization of continuous transverse brachiation gaits.

#### 5.2.1. Single Locomotion Cycle

**Figure 16:**
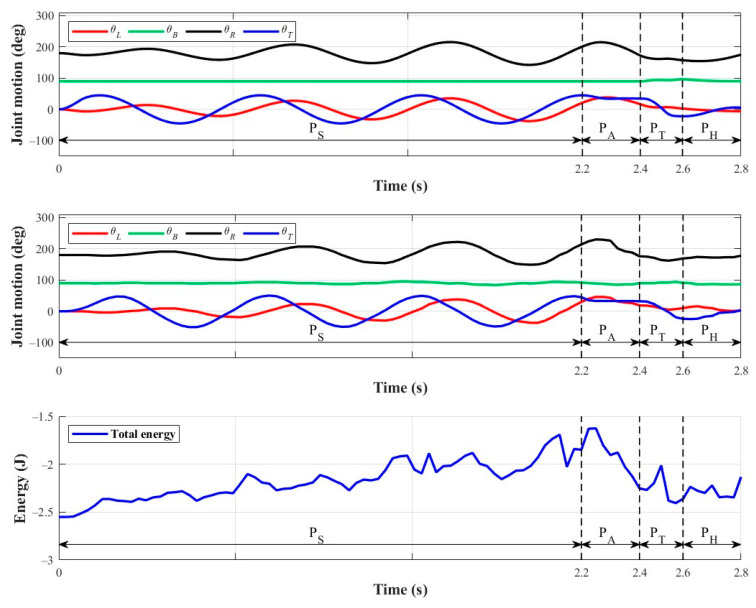
Single locomotion cycle results of transverse ledge brachiation: (**top**) joint trajectories from simulations; (**middle**) joint trajectories from experiments; (**bottom**) energy history from experiments.

**Figure 17:**
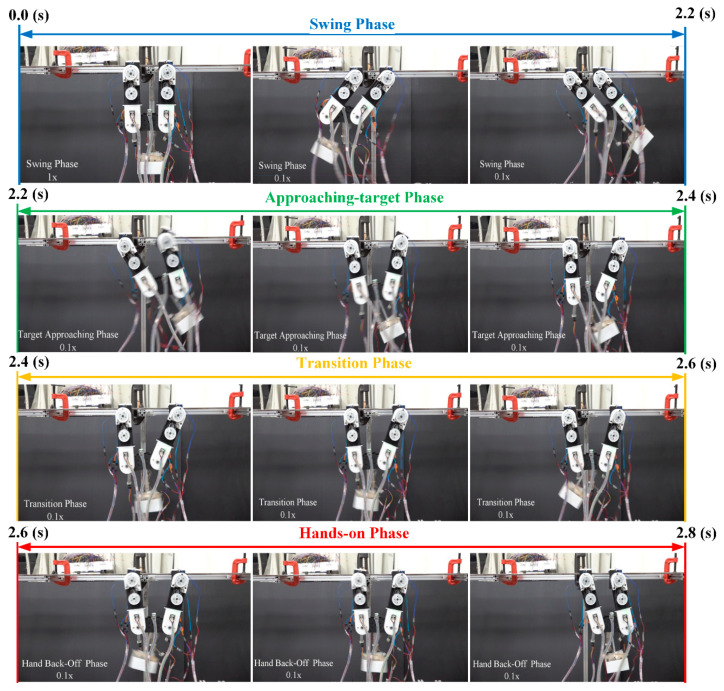
Sequential images of transverse ledge brachiation experiment: a single locomotion cycle.

Figure 16 shows the results of one cycle of the gait. The top two plots show the robot joint trajectories obtained via simulation and experiment, whereas the bottom plot exhibits the changes in total energy throughout the experiment process. Figure 17 shows sequential images of the robot’s motions during the experiment.

Based on the switching conditions obtained in Section 4, the vertical black dashed lines in the graphs in Figure 16 separate the four different movement phases, marked *P_S_*, *P_A_*, *P_T_*, and *P_H_*. First, trends in experimental and simulation results were relatively similar. The body joint angle (green curve) remains at 90° (meaning the body remains in a vertical posture) during most of the process, and the tail joint (blue curve) swings back and forth until the arm joint angles are large enough for the robot to reach out and grasp the ledge. Before the hands-on phase, the left and right joint angles (red and black curves) are coordinated in sync with almost no phase difference, which demonstrates the feasibility and reliability of the proposed model. Next, we calculated the total energy of the entire gait using the following equation and presented the results in the bottom plot of Figure 16:

(15) $$E_{Robot}=T_{Robot}+U_{Robot}$$

In the equation above, *T_Robot_* denotes the kinetic energy of the robot, which can be derived from the total translational and rotational kinetic energy of each link; *U_Robot_* represents the gravity potential energy of the robot, which can be derived using Equation (9). From Figure 16, we can see that excitation and kinetic energy accumulation in the swing phase (*P_S_*) are achieved via the swinging of the lower limb. Thus, the energy in *P_S_* increases gradually with the number of swings. After entering the approaching-target phase (*P_A_*), the system continues the inertia accumulated in the previous phase to perform the transverse motion, so the energy increases at first, but then the overall potential energy swiftly declines due to gravity; thus, the total energy decreases as well. Next, in the posture transition phase (*P_T_*), the swinging of the lower limb first increases the overall kinetic energy and potential energy of the system slightly. However, the range in which the upper limbs can adjust is limited; when the posture of the upper limbs is closer to their mechanical limit, the reduction in the kinetic energy of the robot is even more apparent. Although the potential energy of the system continues to increase, the kinetic energy drops at a greater rate. Thus, the plot shows that initially, the total energy of the system increases and then declines rapidly midway. Nevertheless, it does not return to the initial value when the robot is still. The approaching-target phase (*P_H_*) mainly relies on the left arm returning to its original position to slightly increase the system energy, so the energy does not change significantly in this phase. The trends in the changes in energy during the locomotion process of the robot show that the designed gait can indeed maintain the energy in the various stages, meaning that after the robot begins to swing, its energy does not suddenly drop to the initial value. Thus, there are no sudden stops that cause incoordination in the gait or affect energy utilization efficiency.

To assess the mobility of the robot, we defined the total moving distance of the robot (*M_d_*) under the premise that it is moving to the right, as the final moving distance of the end of the left arm, as shown in Equation (16), where $G_{initial}^{L}$ and $G_{final}^{L}$, respectively, denote the initial and final location of the left gripper. If the left arm of the robot cannot successfully move to the right in the current locomotion cycle, it means that the hands-on phase is not completed and that the moving distance of this locomotion cycle is zero.

(16) $$M_{d}=G_{final}^{L}-G_{initial}^{L}$$

Based on this definition, the moving distances achieved by one locomotion cycle of the transverse brachiation gait were as shown in Table 3; simulation and experimental results were 69 mm and 63 mm, respectively. Aside from modeling errors, this difference may have been caused by the fact that the simulation was established on the assumption of perfect grasping and the fact that the result was obtained from a dynamic model based on a two-dimensional plane. In reality, the robot’s grippers may slip slightly, causing three-dimensional deviations in posture and thereby reducing the moving distance. As it took 2.8 s to complete one locomotion cycle, the movement speed of the robot was 23 mm/s. Based on our experience, if the robot has to stop all motions during a phase in the entire moving process before it can continue to the next phase, then the movement speed achieved would be only about 3 mm/s. This was the case when the gait was not designed with energy continuation in mind. Thus, a design based on energy continuation increases the speed by seven or more times.

#### 5.2.2. Continuous Transverse Brachiation

**Figure 18:**
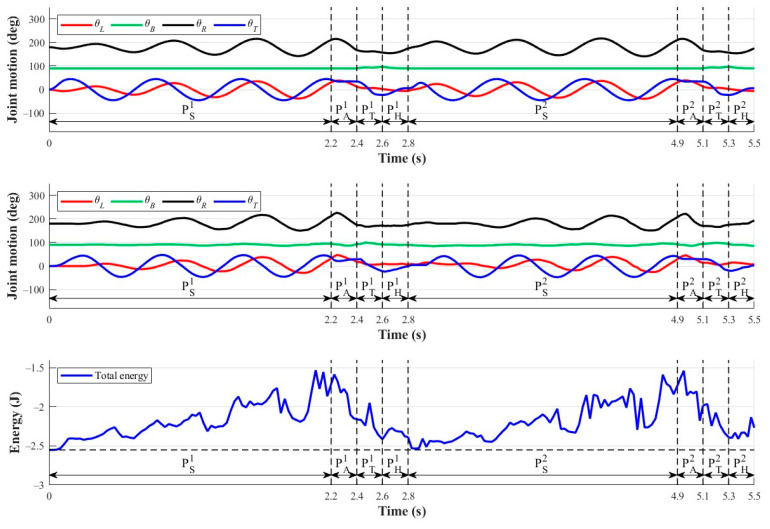
Continuous transverse brachiation for two locomotion cycles: (**top**) joint trajectories from simulations; (**middle**) joint trajectories from experiments; (**bottom**) system energy.

The grasping target of transverse brachiation in this study is a rectangular ledge with boundary conditions in which it is not easy to attain a firm grip. Robots are easily affected by their three-dimensional dynamics, and once it releases the ledge and grabs hold again, the grip posture is generally different from that before releasing the ledge. Thus, after completing one locomotion cycle and beginning the next locomotion cycle, deviations in grip posture may be too great for the same locomotion gait to be successfully achieved. In other words, after the robot completes a few locomotion cycles, poor grip conditions may prevent it from commencing the swing and energy storage phase, or the grippers may gradually slip off the ledge, causing the robot to fall. This is one of the greatest challenges for TLB robots. To explore this interesting problem, we discuss the results of two cycles of transverse brachiation. Figure 18 presents the joint trajectory results. Again, the vertical black dashed lines separate the different movement phases.

The middle plot in Figure 18 shows the second locomotion cycle beginning at 2.8 s, and the amplitudes of the robot’s arm joint angles at the beginning of the swing phase are slightly smaller than those in the first locomotion cycle. It may be that as the arms return to their original positions at the end of the first locomotion cycle and begin the swing phase of the next cycle, the impact dynamics between the grippers and the ledge canceled out some of the system’s energy and affected the robot’s grip posture. This phenomenon can be verified with the total energy changes presented in the bottom plot of Figure 18. In the first cycle, the swinging phase lasted for 2.2 s before accumulating enough energy to begin the subsequent phases. In contrast, the swinging in the second cycle only lasted 2.1 s, but the moving distance resulting from an offset in the initial swinging posture in the second cycle decreased to 58 mm, which was 5 mm shorter than the 63 mm in the first cycle. Nevertheless, the system still retained some energy at the end of the first locomotion cycle that was not completely canceled out by the impact dynamics of the grippers, so there were still some energy continuation effects.

**Figure 19:**
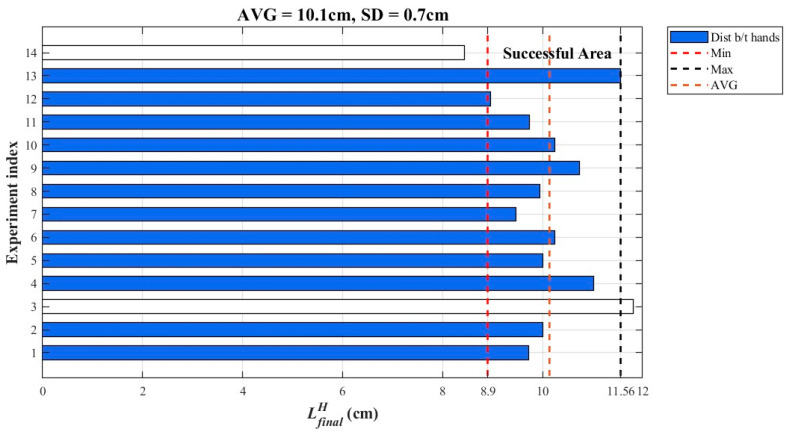
Continuous TLB experiments: the success of implementing the next cycle based on final hand posture in the hands-on phase. White bar indicates failure in experiments.

Experimental results revealed that the final posture of the arms at the end of the hands-on phase is key to whether the next cycle can be achieved successfully. The four-link robot is the most efficient in terms of inertia accumulation when the upper limbs form a parallel four-bar linkage with the ledge. When the posture of the robot’s upper body at the end of the hands-on phase differs greatly from a parallel four-bar linkage, the swinging phase in the next cycle will be unable to accumulate energy successfully. Without this accumulation, the performance in subsequent phases will be negatively affected, and the gait might even fail. Based on the parallel four-bar linkage constraint, we used $L_{final}^{H}$, the distance between the two grippers of the robot at the end of the hands-on phase as an index to analyze the success rate of completing the next cycle. This index also helped us understand the influence of the robot’s posture on continuous transverse brachiation. Figure 19 compiles the results of 14 experiments on two cycles of transverse brachiation, showing the distances between the grippers during the switch from the first to the second cycles. Two experiments failed (meaning the second locomotion cycle could not be executed) and are expressed using white bars; the remaining 12 successful experimental results are presented using blue bars. Theoretically, when $L_{final}^{H}$ equals 10 mm, the width of the body, then the parallel four-bar linkage condition is satisfied, and the success rate of completing the next locomotion cycle will be higher. However, as shown in the figure, only Experiments 2, 5, and 8 came close to this condition at the end of the first locomotion cycle; in most of the experiments, the distance between the two grippers could not satisfy the condition needed for parallel four-bar linkage, but the second locomotion cycle could still be successfully achieved. However, the distance between the two grippers must be the same as the body width for good grip. If swinging or slippage causes any shifts (i.e., the robot’s body is not parallel to the wall as originally assumed), then brachiation may fail. The experimental results showed that robots could complete the second cycle if the distance between the two grippers at the end of the hands-on phase fell between 8.9 cm and 11.56 cm. The mean and standard deviation of $L_{final}^{H}$ were 10.1 cm and 0.7 cm, respectively. With limited degrees of freedom, it is difficult for the robot to maintain a good grip. In this case, the above data can be used to determine whether to execute the next cycle. Without parallel four-bar linkage, reaching for the ledge may result in failure, as in Experiments 3 and 14.

### 5.3. Experiments for Transverse Ricochetal Brachiation

**Figure 20:**
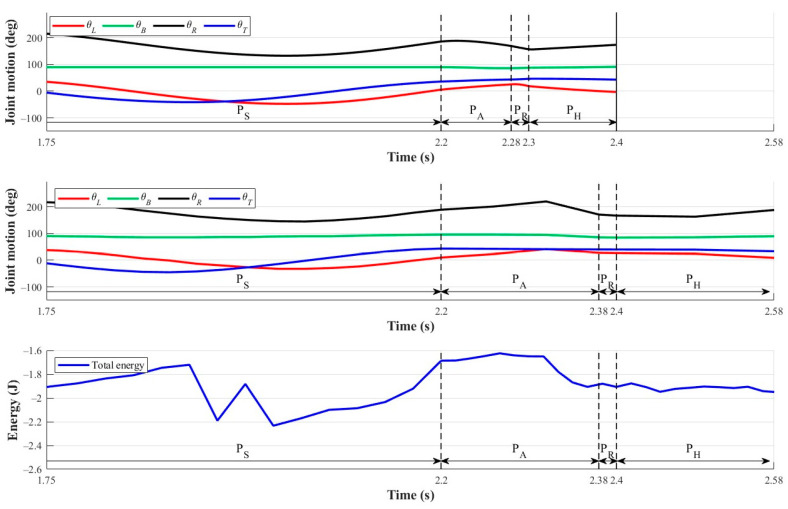
Results of transverse ricochetal brachiation: (**top**) joint trajectories from simulations; (**middle**) joint trajectories from experiments; (**bottom**) energy history from experiments.

**Figure 21:**
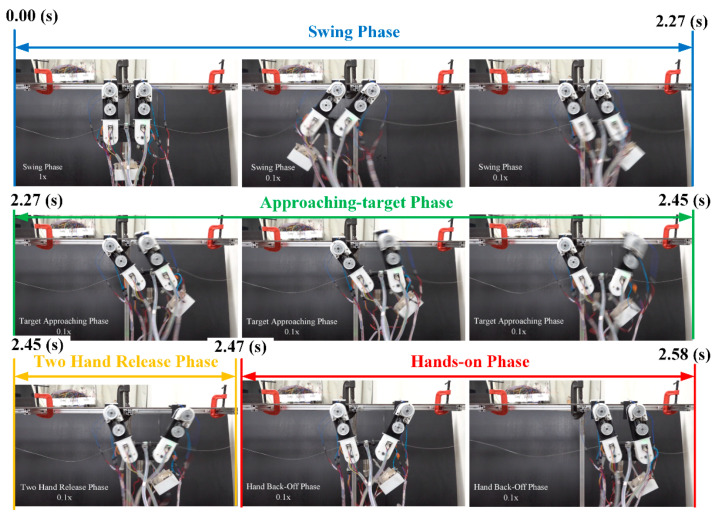
Sequential images of transverse ricochetal brachiation experiment.

Figure 20 and Figure 21 display the simulation results of one TLRB cycle and sequential images of this gait. The sampling rate for simulations was 1 kHz, but due to the IMU readings, the sampling rate for experiments was only 100 Hz. For the latter, changes in total energy were measured by applying the image tracking analysis software Tracker [32] to 60 fps snapshots. For the convenience of presentation, we only show the results midway through the swinging phase in Figure 20.

As can be seen in the figure, simulation and experimental results for the joint trajectories are similar in the swinging phase but differ after the approaching-target phase begins. First, based on the original motion plans and phase-switching condition $\theta_{R}=170^{\circ }$, the simulation indicated that it would take 2.28 s − 2.2 s = 0.08 s to complete the approaching-target phase (*P_A_*), after which the left gripper would release the ledge for the leap motion (i.e., execute *P_R_*). However, in the experiment, it actually took 2.38 s − 2.2 s = 0.18 s to complete the approaching-target phase, thereby creating a delay of 0.18 s − 0.08 s = 0.1 s. In Section 5.2, the approaching-target phase in the TLB experiment was completed in approximately 2.4 s − 2.2 s = 0.2 s as expected. However, in the TLRB experiment, the larger arm motion in the approaching-target phase resulted in a later response. We, therefore, speculate that the dynamic responses of the robot arms may extend the amount of time needed to perform the entire gait due to mechatronic hardware limitations.

**Figure 22:**
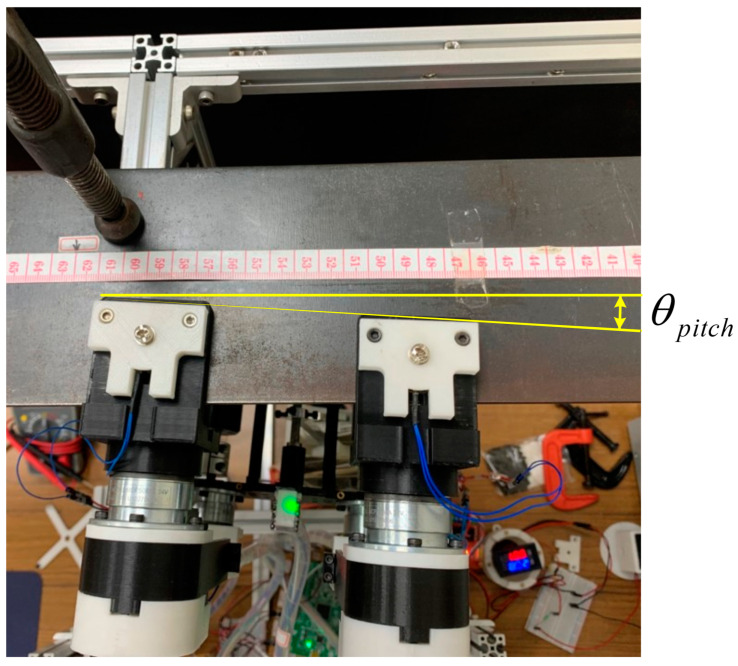
Substantial hand-holding posture deviation ($\theta_{pitch}$) after finishing a locomotion cycle of transverse ricochetal brachiation. Colored lines are added to indicate the deviation angle.

It is not easy to see the leap of the robot in the images in Figure 21. This is because the two-hand-release phase (2.45 s~2.47 s) is closer to and even shorter than the response time of the solenoid electromagnet switches in the grippers. During this phase, the robot’s grippers may still maintain a certain amount of contact with the ledge, which means that the robot’s grippers may slide to the right along the surface of the ledge, thereby increasing the moving distance. Due to the short flight duration (about 0.02 s), we could not accurately determine the time at which the right gripper of the robot grasped the ledge based on the IMU readings. Using trial and error, we had the robot switch to the hands-on phase (*P_H_*) at 2.4 s, and finally, the top view in Figure 22 shows that the robot had obviously turned by an angle of $\theta_{pitch}$ after re-grasping the ledge. With the grip posture of the two grippers already significantly different from the original posture, the robot could not execute the next cycle. Note that such posture deviations were also present within the TLB gait experiment in the previous section, although they were not as prominent. As long as $\theta_{pitch}$ is small enough, the robot can continue to perform swinging excitation. However, these deviations accumulate with each cycle, so only a finite number of locomotion cycles can be achieved. In another aspect, the energy plot shows that because the left gripper releases the ledge for the leap not long after the right gripper grasps the ledge, the reduction in total energy in *P_H_* is only about half or less of the reduction in the *P_H_* of the TLB gait. This demonstrates that during the flight phase, the transverse brachiation robot can more efficiently utilize the energy accumulated in the swing phase to achieve a longer moving distance.

The results above show that due to factors such as hardware limitations and environment dynamics, there are many difficulties in realizing the ideal TLRB gait. Even so, our TLRB experiment still achieved a grasping (moving) distance of 102 mm, which is at least 1.5 times that of the distance achieved by the TLB gait (63 mm). Next, we consider factors affecting the energy consumption of the actuators and analyze gait movement performance using the cost of transport (COT) [33] index, which is defined as follows:

(17) $$\begin{array}{l} \mathrm{COT}≜\frac{E_{a}}{mgd}\\ E_{a}=\int \left|\tau \right|d\theta \end{array}$$

where $E_{a}$ represents the total work performed by the various actuators in the joints of the robot; *m* is the total weight of the robot; and *d* denotes the moving distance of a locomotion cycle. Here, we make a simple comparison of the TLRB and TLB experimental results in Table 4. The TLRB gait indeed consumes more energy (by approximately 30%) but greatly increases the moving distance. Thus, the COT of the TLRB gait has better energy conversion efficiency and movement performance than the TLB gait does, and this is with limitations in the mechatronic hardware of the robot and much of the energy lost during the process due to contact dynamics. From the perspective of bionics, these two gaits are similar to the walking and running gaits of humans. Running consumes more energy than walking and tires the body more easily; however, it is a more efficient mode of locomotion. In another aspect, walking is the safer choice for continuous movement. Humans naturally switch between these two gaits depending on their physical capabilities and changes in the environment to move steadily, continuously, and efficiently. Thus, this study focuses on a means of enabling transverse brachiation robots to switch to a more suitable locomotion gait depending on the circumstances. Please refer to the following link for a video from our experiments: https://youtu.be/j11oPRQxvqA (accessed on 14 May 2023).

## 6. Conclusions

This study presents the locomotion design and implementation of an arm-body-tail ledge brachiation robot mimicking the transverse movements of climbing athletes. Under the constraint of two pairs of parallel links, we consider the cause-and-effect relationship between the designed phases to identify phase-switching conditions and desired joint motion. Experiments demonstrate that locomotion without leaping action can be successfully conducted for two consecutive cycles, and the locomotion of ricochetal brachiation represents a significant improvement in terms of grabbing distance and energy efficiency. However, the experiments all terminated during the locomotion process due to deviation in the hand-holding postures; this impeded energy accumulation in the next cycle and greatly increased the risk of falling. Future research could investigate landing posture dynamics using improved mechatronic design and three-dimensional modeling. Active posture compensation methods and advanced robot locomotion control could further improve the safety and robustness of transverse ledge brachiation.

## Figures and Tables

**Table 1:** Comparison of bio-inspired transverse ledge brachiation/climbing robots.

	Current Study	Transverse Ledge Ricochetal Brachiation Robot [1] (Lin and Yang, 2020)	Transverse Ledge Climbing Robot [10] (Lin and Hu, 2022)	Transverse Ledge Brachiation Robot [2] (Lin and Tien, 2022)
Number of robot link	4	4	5	4
Robot configuration	Arm-body-tail	Arm-body-tail	Arm-body	Arm-body-tail
System classification	Arm-body-tail	Arm-body-tail	Arm-body	Arm-body-tail
Multi-locomotion	Yes (TLB/TLRB)	No (TLRB)	No (TLC)	No (TLB)
Application scenario	Horizontal brachiation (continuous/ gapped ledges)	Horizontal brachiation (continuous/gapped ledges)	Horizontal climbing (continuous/sloped ledges)	Horizontal and non-level horizontal brachiation (ledges at different elevations)
Continuous brachiation (via swing)	Yes	No	N/A	No
Lower limbs	Yes	Yes	No	Yes

**Table 2:** Robot model parameters.

Symbol	Physical Meaning	Symbol	Physical Meaning
$m_{L}$	Center of mass of left arm	$l_{RC}$	Distance between right wrist joint and center of mass of right arm
$m_{R}$	Center of mass of right arm	$l_{BC}$	Distance between left shoulder joint and center of mass of body
$m_{B}$	Center of mass of body	$l_{LC}$	Distance between left wrist joint and center of mass of left arm
$m_{T}$	Center of mass of tail	$l_{T}$	Tail length
$I_{L}$	Moment of inertia of left arm	$l_{BW}$	Body width
$I_{R}$	Moment of inertia of right arm	L	Distance between two grippers
$I_{B}$	Moment of inertia of body	$\theta_{L}$	Joint angle of left arm
$I_{T}$	Moment of inertia of tail	$\theta_{R}$	Joint angle of right arm
$l_{L}$	Length of left arm	$\theta_{B}$	Body joint angle
$l_{R}$	Length of right arm	$\theta_{T}$	Tail joint angle

**Table 3:** Motion performance for transverse ledge brachiation.

	Simulation	Experiment
Grabbing distance at phase *P_H_* (mm)	69	63
Time to finish a locomotion cycle (s)	2.8	2.8
Movement speed (mm/s)	24.6	22.5

**Table 4:** Comparison of proposed two robot locomotion styles.

	Transverse Ledge Brachiation	Transverse Ledge Ricochetal Brachiation
Grabbing distance (mm)	63	102
Time (s)	2.8	2.58
Movement speed (mm/s)	22.5	39.5
Robot weight (kg)	1.76	1.76
Actuator energy (J)	6.15	8.02
COT (J·kg^−1^·m^−2^·s^2^)	5.65	4.56

## Data Availability

Not applicable.
